# A most wanted list of conserved microbial protein families with no known domains

**DOI:** 10.1371/journal.pone.0205749

**Published:** 2018-10-17

**Authors:** Stacia K. Wyman, Aram Avila-Herrera, Stephen Nayfach, Katherine S. Pollard

**Affiliations:** 1 Gladstone Institutes, San Francisco, CA, United States of America; 2 University of California, Berkeley, CA, United States of America; 3 Lawrence Livermore National Laboratory, Livermore, CA, United States of America; 4 University of California, San Francisco, CA, United States of America; 5 DOE Joint Genome Institute, Walnut Creek, CA, United States of America; 6 Chan-Zuckerberg Biohub, San Francisco, CA, United States of America; CPERI, GREECE

## Abstract

The number and proportion of genes with no known function are growing rapidly. To quantify this phenomenon and provide criteria for prioritizing genes for functional characterization, we developed a bioinformatics pipeline that identifies robustly defined protein families with no annotated domains, ranks these with respect to phylogenetic breadth, and identifies them in metagenomics data. We applied this approach to 271 965 protein families from the SFams database and discovered many with no functional annotation, including >118 000 families lacking any known protein domain. From these, we prioritized 6 668 conserved protein families with at least three sequences from organisms in at least two distinct classes. These Function Unknown Families (FUnkFams) are present in Tara Oceans Expedition and Human Microbiome Project metagenomes, with distributions associated with sampling environment. Our findings highlight the extent of functional novelty in sequence databases and establish an approach for creating a “most wanted” list of genes to prioritize for further characterization.

## Introduction

Genome sequencing and metagenomics are producing unprecedented amounts of data but elucidation of gene function has not kept pace with the volume of identified genes. Homology-based annotation methods predict domains and functions for many new protein coding and RNA genes. However, many sequenced genes do not have significant homology to experimentally characterized domains or gene families [1]. To quantify this problem, we developed a bioinformatics approach to identify *bona fide* protein families with no annotation and then characterized these with respect to their phylogenetic range and abundance in metagenomes. The result is FUnkFams, a prioritized catalog of genes for experimental discovery of function.

## Methods

### FUnkFams construction

Our pipeline of custom scripts begins with protein families. We first drop families with too few unique protein sequences (<3 in this study) and families where >50% of the sequences lack a start or stop codon. This rigorous filtering eliminates some small families but helps to identify *bona fide* families of full-length proteins. We then search for all the proteins in these families in annotation databases to annotate domains in every sequence. These database searches are designed to identify the exact protein (100% identical hit over the full length of the protein sequence using a blastp search with default parameters), not to identify homologs. The rationale for this strategy is that the protein families in this study derive from genomes that have been processed into the databases, and hence any proteins from these genomes should have been annotated already based on homology and other criteria of the databases. The 100% exact match criterion could be changed to search for homologs if using protein families derived from metagenomes or other sources that may not be in the annotation databases. Next, we characterize each sequence in each protein family according to the NCBI taxonomic annotation of the genome from which it derived and then quantify how many different species, genera, families, orders, classes, phyla, kingdoms, and domains are represented in each gene family.

### Profiling in metagenomes

We used Diamond [2] to align reads from the Human Microbiome Project (HMP)[3] and Tara Oceans (TO)[4] metagenome samples to a database of protein family sequences. We counted aligned reads for each family, requiring a best hit to a protein belonging to the family with at least 99% DNA sequence identity over the whole length of the read. Families with at least one read count were called present in the metagenome. Family abundance in each sample was estimated using reads per kilobase of genome (RPKG), a statistic that normalizes for both protein family length (mean of all member sequences in database) and average genome size (estimated from the metagenomics sample with MicrobeCensus) [5].

### Association testing in HMP

We tested for association between a number of host phenotypes and protein family presence in HMP metagenomes. We investigated associations with 13 host phenotypes that reflect lifestyle and medication use, as defined in HMP documentation. Phenotype data was obtained with permission through dbGaP (study ID = phs000228.v2.p1). Phenotypes were required to have at least two values with more than four observations. Seven subject variables passed this filtering step: bmi category, contraceptive use, breastfed status, diet, education level, birth country and student status. We fit a logistic regression model for each protein family and used the resulting coefficients and their standard errors to perform t-tests to identify phenotypes associated with the presence of each family across samples from each body site. The models account for geographic location (SITE variable in HMP) and were fit for each body site. P-values were corrected for multiple testing using the false discovery rate (FDR). We repeated this analysis within body subsites using the same filtering criteria.

### Association testing in tara oceans data

Environmental data was downloaded from the Tara Oceans data resource (http://ocean-microbiome.embl.de). We fit logistic regression models for protein family presence versus environmental variables, adjusting for latitude and month. Separate models were fit for samples collected with each filter size (size fraction). The resulting t-test p-values were adjusted for multiple testing using FDR.

## Results

### Identifying full-length proteins with no annotated domains

**Fig 1:**
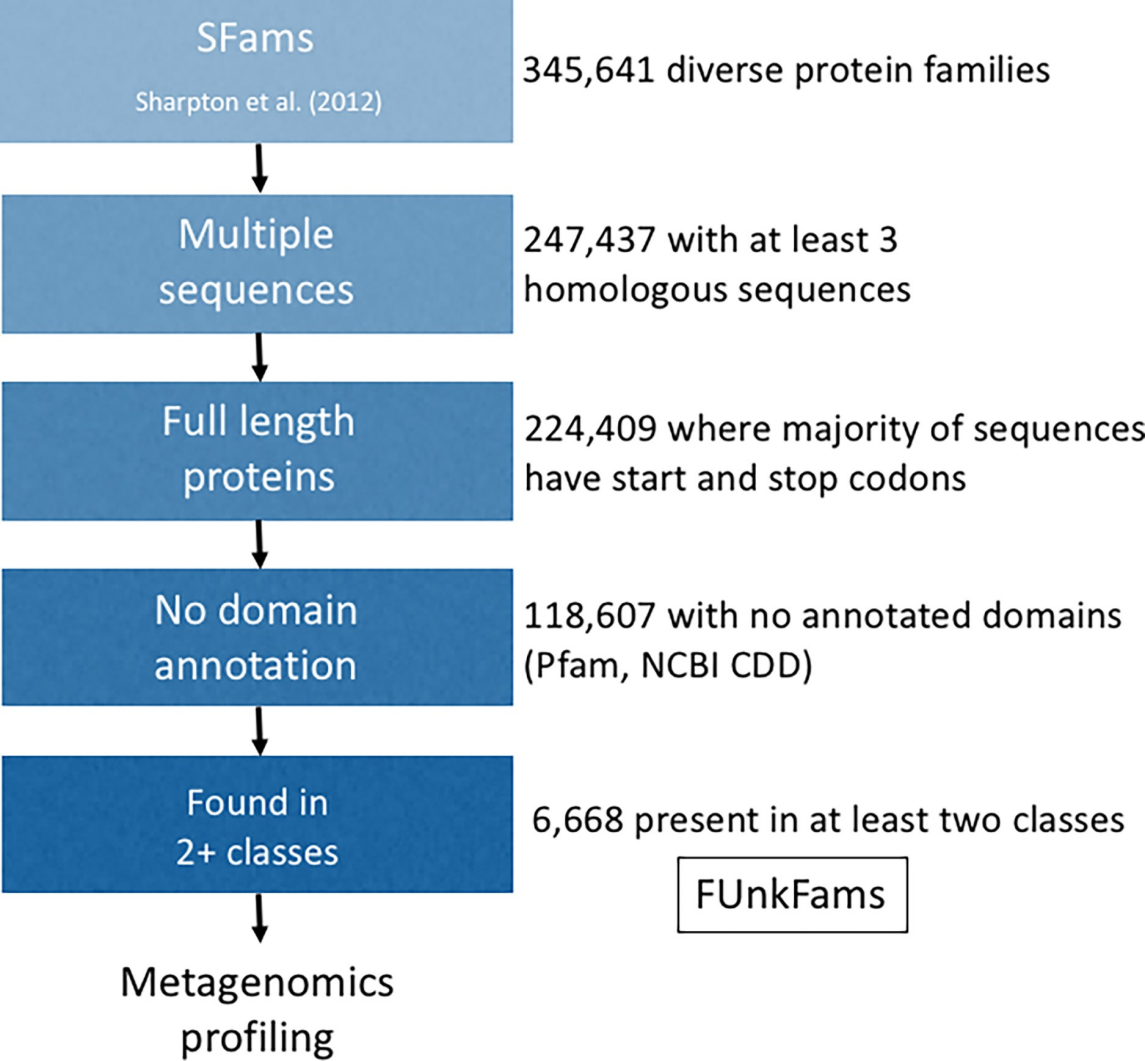
Bioinformatics pipeline for identifying FUnkFams from the SFams database.

We built a bioinformatics pipeline (Fig 1) that begins with a database of gene families, filters out truncated sequences without a start and stop codon, assigns annotations to all sequences in each family using one or more annotation databases, and records the taxonomy of the organism from which each sequence derived (Methods). In a second step, metagenomic sequencing reads from two large, publicly available collections of samples are mapped to protein families, resulting in an estimate of protein family abundance in each sample. These data are then used to organize and rank gene families based on their level of annotation, number of sequences, phylogenetic diversity, and distribution across metagenomes.

**Fig 2:**
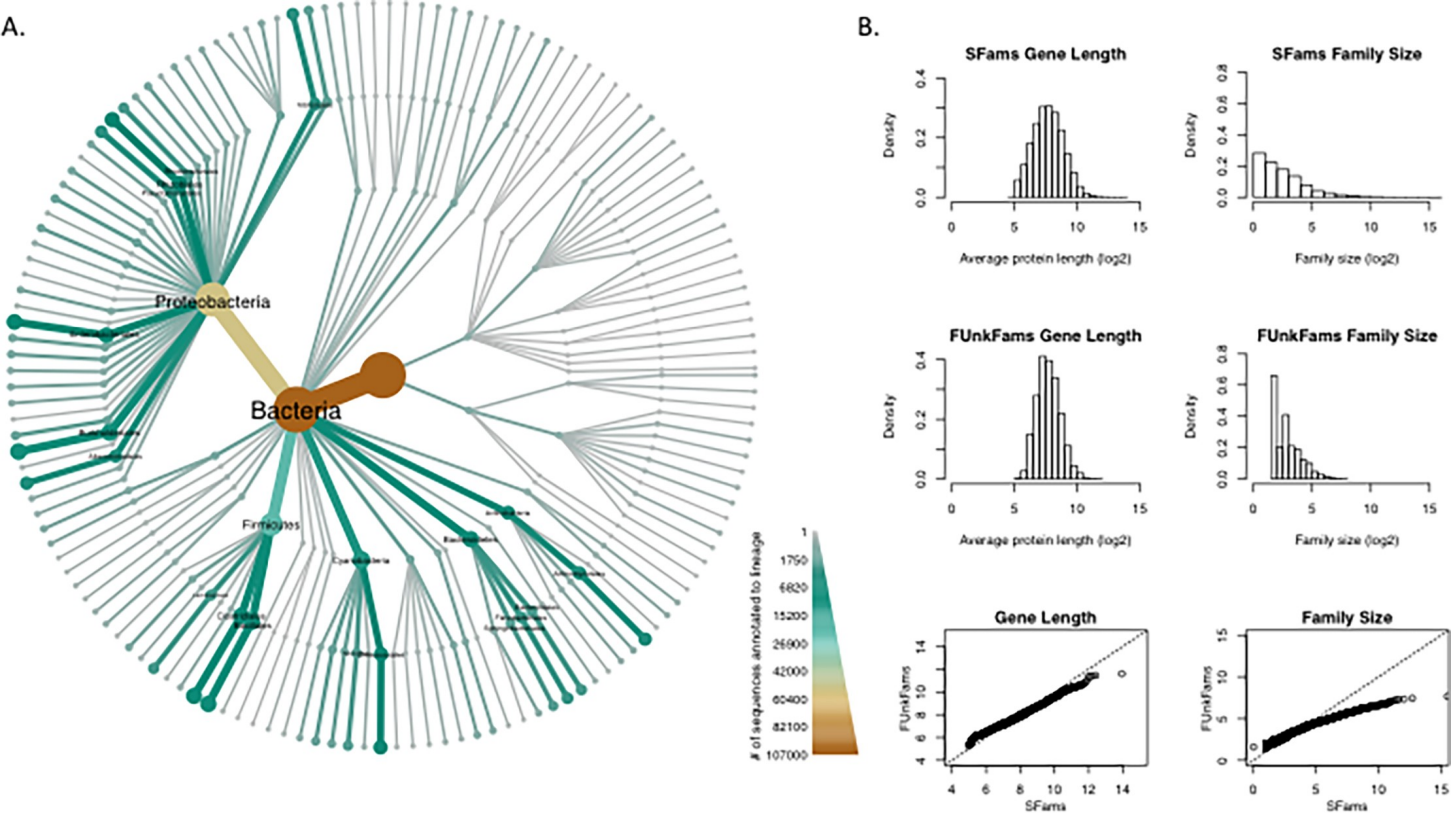
Phylogenetic distribution, family size, and sequence length of FUnkFams. (A) Phylogenetic heat tree of proteins in FUnkFams generated with Metacoder [10]. Each FUnkFams protein sequence was annotated with the taxonomic label of the genome from which it was derived. The color of a branch represents the number of proteins from any FUnkFam on that branch of the taxonomy. The tree shows that FUnkFams are present across diverse lineages of cellular organisms including families from all three domains and over thirty phyla. Proteobacteria contribute many sequences to FUnkFams, in part because many genomes have been sequenced from that phylum. We also generated a heat tree of all SFams, illustrating lineages where FUnkFams are enriched given how many genomes have been sequenced (S2 Fig). (B) FUnkFams protein length (in amino acids, log2 scale) and family size (number of protein sequences) are comparable to other SFams. Top and middle panels show histograms, and bottom panels are quantile-quantile plots showing that most quantiles of length and size are equal between FUnkFams and SFams, except at the top quantiles where SFams are slightly longer (i.e., more amino acids) and bigger (i.e., more sequences).

We applied this approach to discover the least annotated, most phylogenetically diverse full-length protein families in the SFams database [6]. SFams are a set of protein families generated by iterative clustering of over ~10.5 million protein sequences from over 3000 references genomes based on sequence homology. We used SFams because it was compiled in a comprehensive, automated fashion from thousands of diverse genome sequences, and we applied bioinformatics filters to remove small and truncated families and possible artifacts. Specifically, we first identified 224 409 SFams with at least three unique, homologous, full-length protein sequences (Fig 1). We then annotated the sequences in these SFams using two curated and frequently updated sources of protein domains: the PFam database [7] and the NCBI Conserved Domain Database (CDD) [8]. Of the many protein database choices, we chose these two because they are persistent, curated, and updated, while others tend to be transient, uncurated and propagate annotation errors from other databases. SFams were identified in PFam and CDD using blastp exact matches to any of the sequences in the SFam, which answers the question of whether any member of the protein family has any annotated domains (already identified via homology by these databases) and is not an attempt to annotate the protein family (which would use non-exact matches). This analysis showed that the majority of protein families lack even a single domain annotated in PFam or CDD (N = 118 607 SFams, 52.9% of total). These protein families without domain annotation are comprised of sequences from many branches of the cellular tree of life (Fig 2A). For further analysis and prioritization we selected a subset of 6 668 protein families with no annotated domains and sequences from two or more taxonomic classes (S1 Fig). We call these Function Unknown Families (FUnkFams)(S1 Table). Of these, most FUnkFams (84.3%) are not in UniProt xref database [9], and those that are in UniProt (N = 1 045) are largely annotated as hypothetical or uncharacterized proteins (S2 Table), with just eight FUnkFams containing a sequence that has an xref-annotated function, despite having no domain annotation (S5 Table).

FUnkFams are similar to other SFams in terms of properties other than the criteria we used to define them (i.e., functional annotation and phylogenetic breadth). Protein sequences in FUnkFams have a similar phylogenetic distribution to all SFams (S1 Fig) with some enrichment in Cyanobacteria. They are also somewhat depleted in eukaryotes and archaea, probably due to bacterial SFams being more likely to meet our criteria of multiple homologous sequences from at least two classes. Like SFams, a typical FUnkFam is approximately 250 amino acids long (Fig 2B) and is comprised of three to five sequences (Fig 2C), though FUnkFams are slightly depleted for very long and very large families compared to better-annotated SFams. Nonetheless, six FUnkFams are comprised of more than 100 sequences, including a Proteobacterial family (SFams.ID = 4560) with 203 sequences and a family (SFams.ID = 5980) with 145 sequences spanning multiple domains of life. Thus, FUnkFams appear to be representative of full-length, phylogenetically diverse protein families.

### Profiling FUnkFams with shotgun metagenomes

To investigate the ecological distributions of FUnkFams, we quantified their abundance in shotgun metagenomes from the Tara Oceans Expedition (TO; 243 samples from 210 ecosystems in 20 biogeographic provinces at different depths over the course of three years) [4] and Human Microbiome Project (HMP; 699 samples from oral, airways, skin, gut, vaginal sites on 300 healthy individuals at up to three time points over two years) [3] (Methods). To pre-filter FUnkFams without sufficient variation in presence across samples to detect associations, we only included FUnkFams with entropy in the top 25th percentile. To focus on the most phylogenetically diverse FUnkFams, we additionally only included those with sequences derived from genomes in at least two phyla. This filtering resulted in 319 FUnkFams for HMP and 100 for TO.

**Fig 3:**
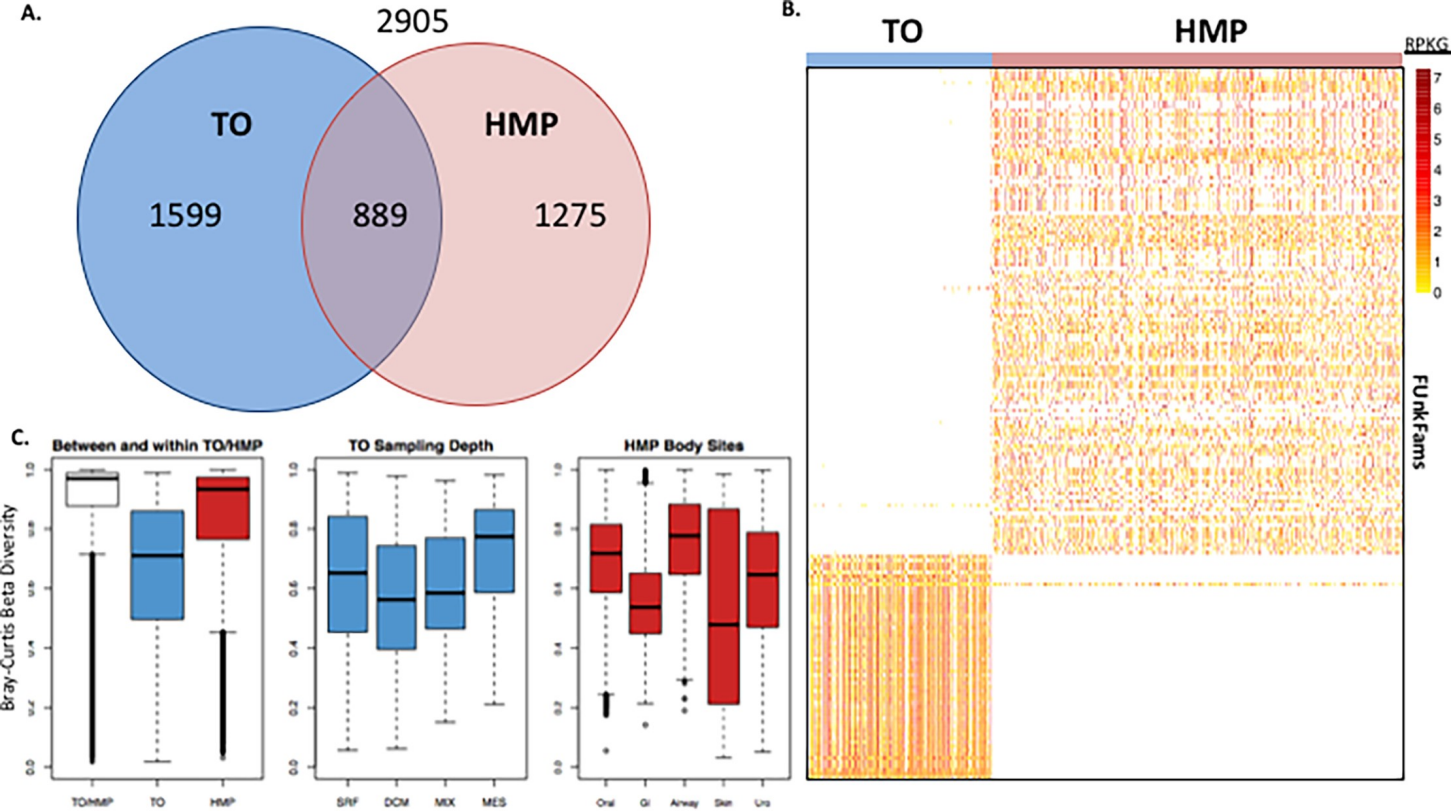
FUnkFams are present in marine and human metagenomes. (A) Most FUnkFams are detected in either TO or HMP metagenomes (56.6%), but relatively few are present in both environments (13.3%). (B) Heatmap showing the abundance of FUnkFams (rows) in TO (left) or HMP (right) metagenomes after normalizing for gene length, library size, and average genome size (RPKG—reads per kb of gene sequence per genome equivalent [5]). The 180 FUnkFams with at least 50 aligned reads across all samples are displayed (see S4 Fig for the unfiltered heatmap of all FUnkFams). (C) Distributions of Bray-Curtis dissimilarity between pairs of samples from marine environments (TO; blue), between pairs of samples from human microbiomes (HMP; red), and between pairs of samples from different environments (white). Bray-Curtis dissimilarity is a measure of the compositional dissimilarity between two populations, where a value of 1 means the they share no species and 0 means they share all species. Samples are more similar within than between the two environments. SRF, surface water; DCM, deep chlorophyll maximum; MIX, mixed layers; MES, mesopelagic.

The majority of FUnkFams (56.6%) are present in at least one of these 942 metagenomes, with many detected in multiple metagenomes (32.5% in at least two HMP samples, 37.2% in at least two TO samples) but relatively few (13.3%) detected in both TO and HMP (Fig 3A). FUnkFam prevalence was generally higher in TO (mean = 18.6% versus 8.1%), with TO samples averaging 700 detected FUnkFams and HMP averaging 304 (S2 Fig). Higher sequencing depth in TO may contribute to this signal. A particularly prevalent set of 137 FUnkFams was found in over 90% of TO samples, while just three were in over 90% of HMP samples, likely reflecting greater annotation of functions found in the human body samples relative to marine samples but also potentially also due to ecological differences between human body sites. Abundance of detected FUnkFams is on average higher in TO, though many FUnkFams are approximately equally abundant between TO and HMP (Fig 3B and S2 Fig) and 27 are highly abundant in both environments (S3 Fig). Reflecting the ecological specificity of many FUnkFams, beta-diversity is significantly higher between the two environments than between samples within either environment (Fig 3C).

We next used logistic regression to quantify how these differences in FUnkFam distributions across TO and HMP correlate with characteristics of the samples after adjusting for technical variables (Supplemental Methods). In TO, the presence of three FUnkFams was significantly associated with nitrate level after multiple testing correction (FDR<5%). One of these FUnkFams was also significantly associated with salinity and longitude, and another was significantly associated with longitude, latitude, temperature, and depth (S3 Table). Other FUnkFams showed weaker associations with environmental variables (S4 and S5 Figs). The dominant variable associated with FUnkFam presence in HMP samples is body site (S4 and S6 Figs; S4 Table), with only a few FUnkFams broadly detected across body sites. Other host phenotypes, such as BMI, smoking status, or diet, were not significantly associated with the presence of any FUnkFams.

## Conclusions

These results identify thousands of uncharacterized protein families composed of homologous sequences from phylogenetically diverse organisms that are abundant in the human body or global oceans. These characteristics suggest that FUnkFams are *bona fide* protein families, and the associations of specific FUnkFams with marine environments or body sites provide hints about protein function and ecology. FUnkFams constitute a “most wanted” list for protein families with no known domains, because they have so little annotation but are made up of multiple, phylogenetically diverse, full-length protein sequences that are frequently detectable in metagenomes. Functionally characterizing these gene families would broaden our understanding of the genomes and environments in which they are found. This study therefore lays the groundwork for significant future work to (i) predict (e.g., via genome proximity and further metagenome profiling [11] or literature based similarity [12]) and (ii) experimentally validate (e.g., via biochemical and structural characterization [13]) the functions of FUnkFams and the unannotated protein domains they contain. Identifying annotated proteins with distant homology to FUnkFams or recently sequenced homologs that are not in the SFams database could help determine what functional assay (e.g., enzyme kinetics versus DNA binding) would be most useful for each family. Our approach can be flexibly extended to use other databases of gene families and sources of functional annotation, and it will be interesting to apply it to other protein catalogs as well as RNA genes.

Supplementary information is available at the Journal’s website. FUnkFams data are freely available via figshare at: https://figshare.com/projects/Function_Unknown_Families_of_homologous_proteins_FUnkFams_/25924.

## Supporting information

S1 Fig (A) Number of FUnkFams found across multiple domains, phyla, and classes in the tree of cellular organisms (e.g. 208 FUnkFams were found across more than one domain). (B) Metacoder phylogenetic heat tree of SFams abundance across cellular organisms. Color indicates number of sequences on a branch. A random subset of 400 000 SFams was used to generate the tree. (C) Metacoder phylogenetic heat tree of FUnkFams abundance across cellular organisms (as in Fig 1A, for comparison here with SFams tree).
(TIFF)

S2 Fig (A) Prevalence (vertical axis) of FUnkFams in TO (blue) and HMP (red) samples, ordered by decreasing prevalence in HMP (horizontal axis). (B) Prevalence (vertical axis) of FUnkFams in TO (blue) and HMP (red) samples, ordered by decreasing prevalence in TO (horizontal axis). Many FUnkFams are more prevalent in TO than HMP, but the converse is not true. (C) For 889 FUnkFams present in at least one TO and at least one HMP sample, the fractional abundance (vertical axis) represents the proportion of total RPKG for the FUnkFam that comes from TO (blue) versus HMP (red). FUnkFams are ordered by decreasing proportion of total RPKG deriving from TO samples (horizontal axis).
(TIFF)

S3 Fig Heatmap with all 3 763 FUnkFams (rows) detected in any metagenome (TO, HMP or both) at any abundance. Blue (left columns) are TO samples and red (right columns) are HMP samples.
(TIFF)

S4 Fig PCA plots of samples from HMP (A-B) and TO (C-E) based on counts of metagenomic sequencing reads mapped to all FUnkFams. HMP samples cluster by body site (A) but not other phenotypes such as BMI (B). TO samples cluster by marine layer (E) but not other environmental features (C-D)
(TIFF)

S5 Fig Heatmap for most abundant FUnkFams in TO samples, clustered both by column (samples) and row (FUnkFams) with environmental features annotated across rows. (TIFF)

S6 Fig Heatmap for most abundant FUnkFams in HMP samples, clustered both by column (samples) and row (FUnkFams) with host phenotypes annotated across rows. (TIFF)

S1 Table Characteristics of FUnkFams, including phylogenetic distribution and prevalence in TO and HMP samples. (XLSX)

S2 Table Annotations for 1 045 FUnkFams with a protein sequence in the UniProt xref database. (XLSX)

S3 Table Results of statistical tests for associations between environmental variables and FUnkFams presence across TO samples. (XLSX)

S4 Table Results of statistical tests for associations between host phenotype variables and FUnkFams presence across HMP samples. (XLSX)

S5 Table Annotations for eight FUnkFams with a protein sequence whose function is annotated in the UniProt xref database (despite having no annotated domains). (XLSX)
