# Supplementary figures and images for: A most wanted list of conserved microbial protein families with no known domains
Source: PLoS One. 2018 Oct 17;13(10):e0205749. doi: 10.1371/journal.pone.0205749 (PMC6192648; doi:10.1371/journal.pone.0205749)

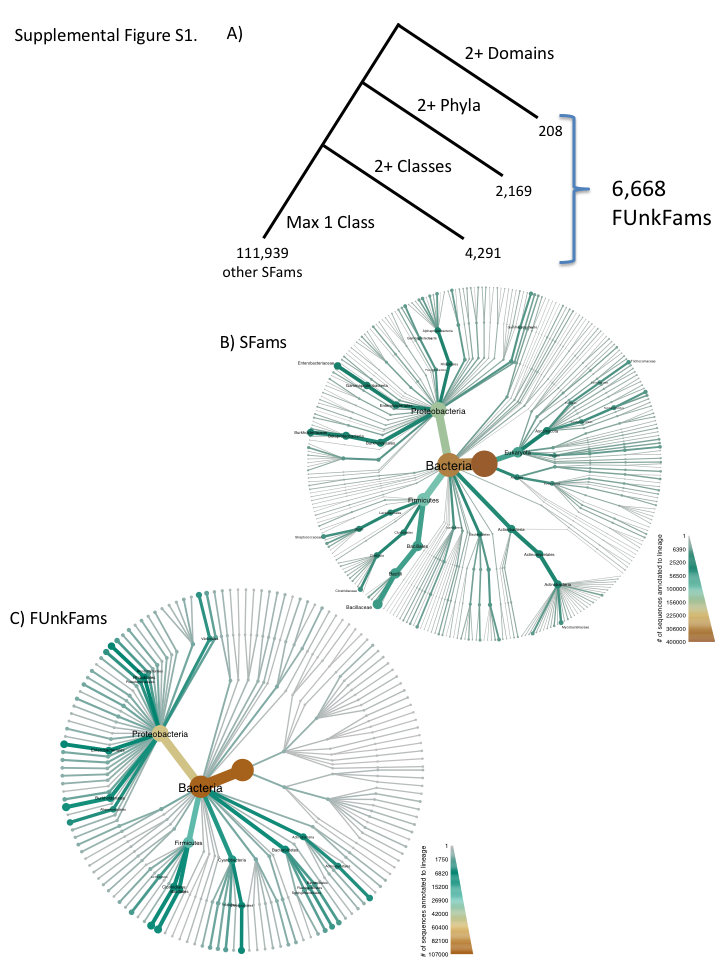

Supplement: S1 Fig — (A) Number of FUnkFams found across multiple domains, phyla, and classes in the tree of cellular organisms (e.g. 208 FUnkFams were found across more than one domain). (B) Metacoder phylogenetic heat tree of SFams abundance across cellular organisms. Color indicates number of sequences on a branch. A random subset of 400 000 SFams was used to generate the tree. (C) Metacoder phylogenetic heat tree of FUnkFams abundance across cellular organisms (as in Fig 1A, for comparison here with SFams tree). (TIFF) [file pone.0205749.s001.tiff]

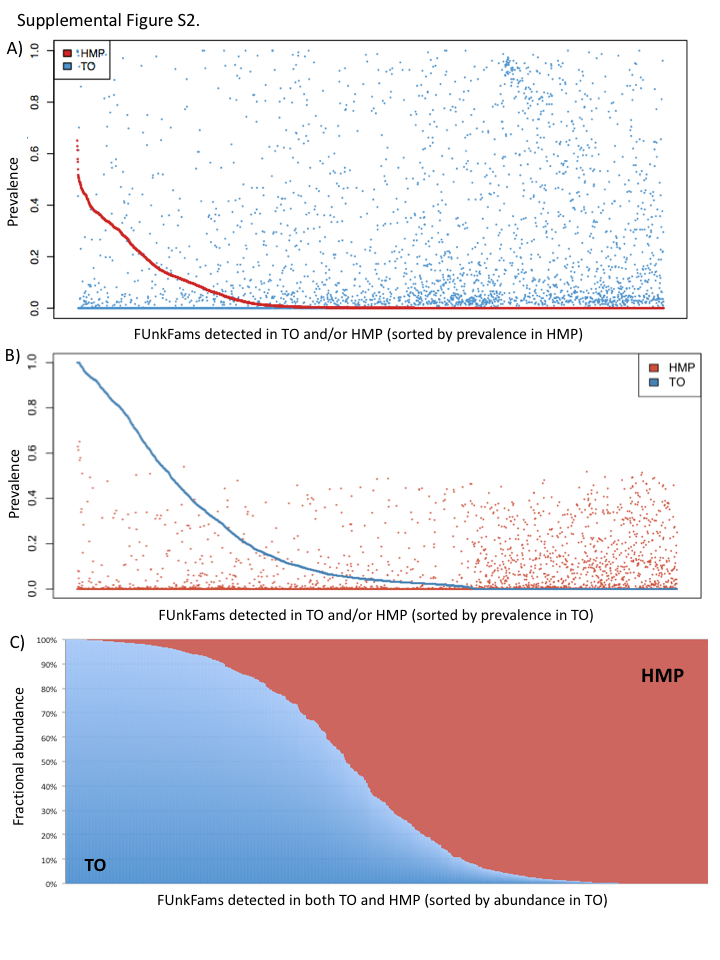

Supplement: S2 Fig — (A) Prevalence (vertical axis) of FUnkFams in TO (blue) and HMP (red) samples, ordered by decreasing prevalence in HMP (horizontal axis). (B) Prevalence (vertical axis) of FUnkFams in TO (blue) and HMP (red) samples, ordered by decreasing prevalence in TO (horizontal axis). Many FUnkFams are more prevalent in TO than HMP, but the converse is not true. (C) For 889 FUnkFams present in at least one TO and at least one HMP sample, the fractional abundance (vertical axis) represents the proportion of total RPKG for the FUnkFam that comes from TO (blue) versus HMP (red). FUnkFams are ordered by decreasing proportion of total RPKG deriving from TO samples (horizontal axis). (TIFF) [file pone.0205749.s002.tiff]

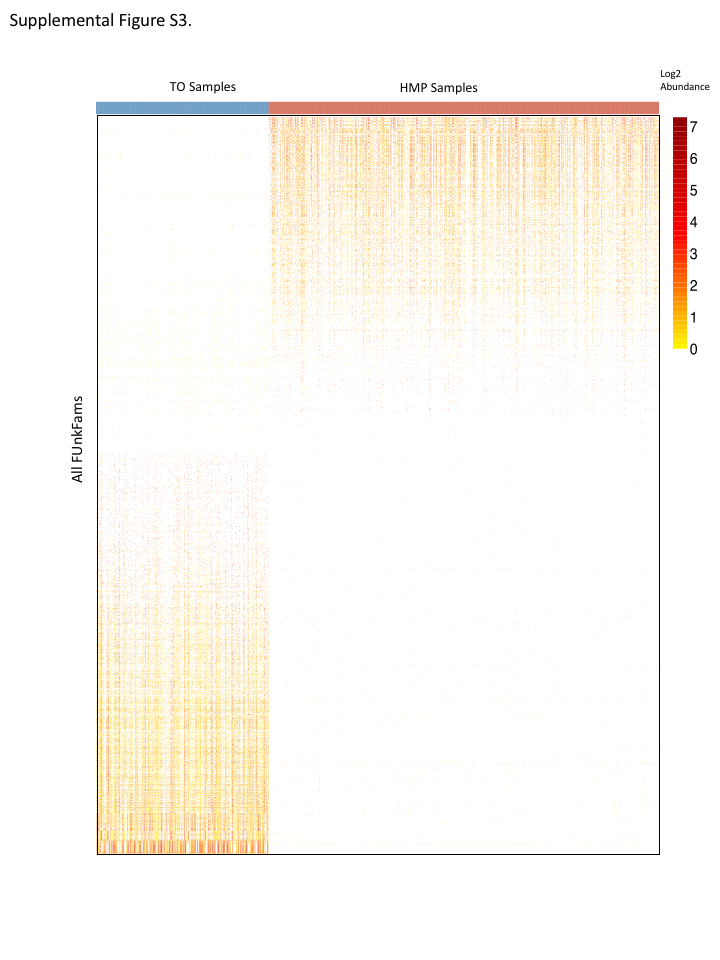

Supplement: S3 Fig — Blue (left columns) are TO samples and red (right columns) are HMP samples. (TIFF) [file pone.0205749.s003.tiff]

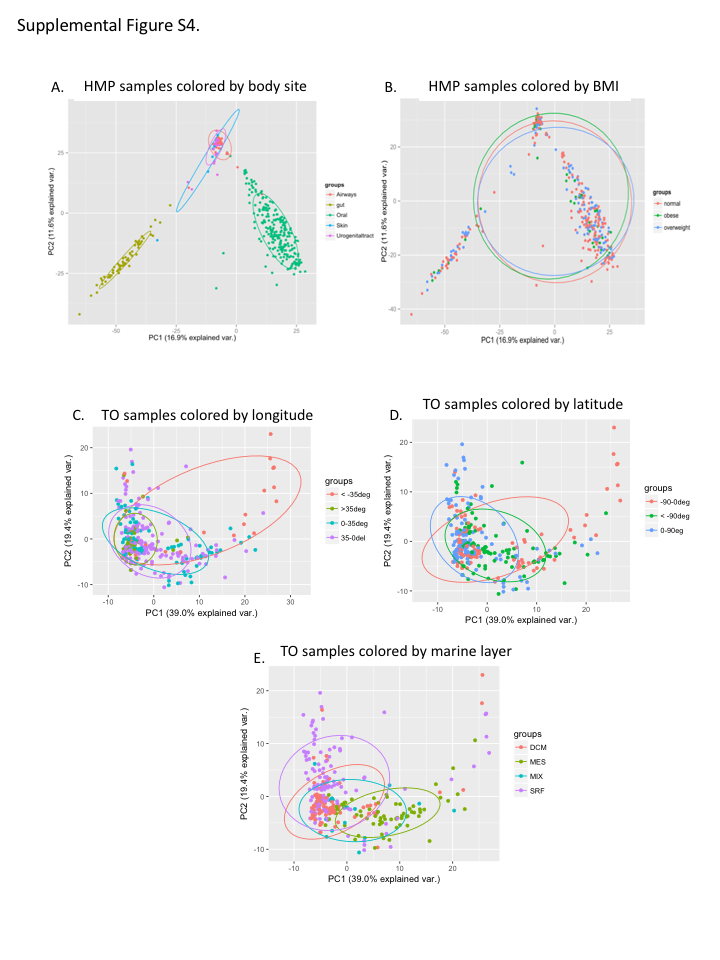

Supplement: S4 Fig — PCA plots of samples from HMP (A-B) and TO (C-E) based on counts of metagenomic sequencing reads mapped to all FUnkFams. HMP samples cluster by body site (A) but not other phenotypes such as BMI (B). TO samples cluster by marine layer (E) but not other environmental features (C-D) (TIFF) [file pone.0205749.s004.tiff]

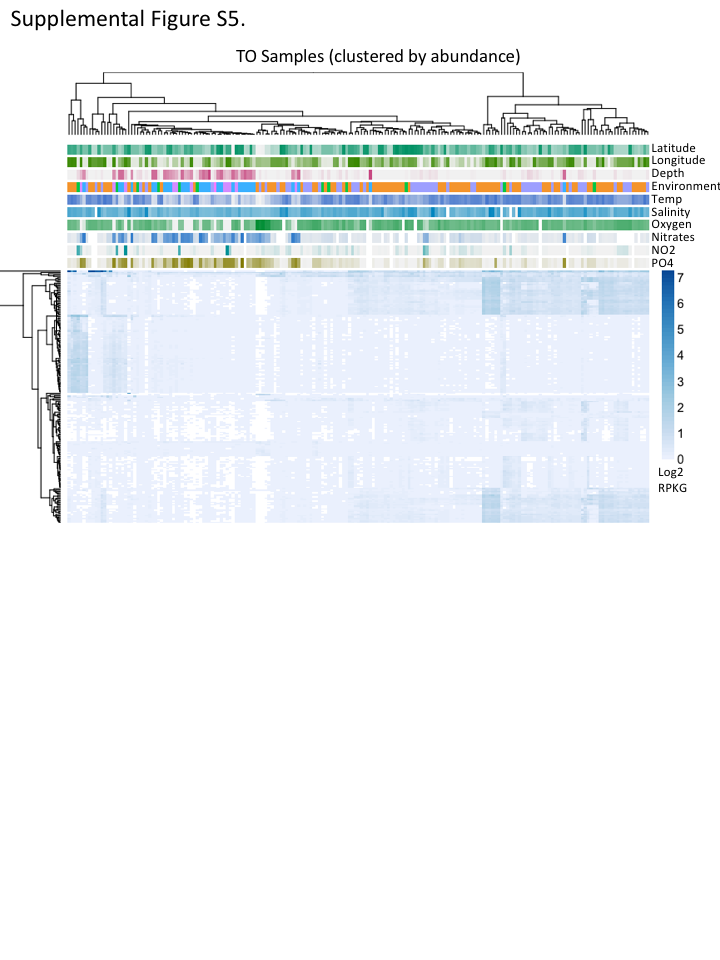

Supplement: S5 Fig — (TIFF) [file pone.0205749.s005.tiff]

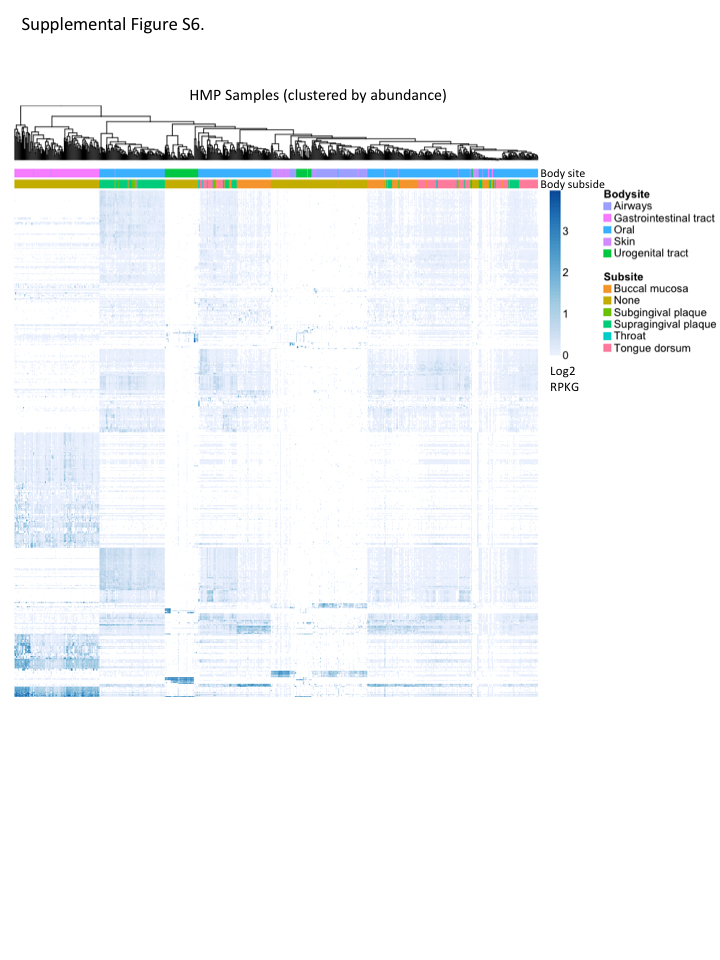

Supplement: S6 Fig — (TIFF) [file pone.0205749.s006.tiff]
